# Understanding metabolic alterations after SARS-CoV-2 infection: insights from the patients’ oral microenvironmental metabolites

**DOI:** 10.1186/s12879-022-07979-y

**Published:** 2023-01-23

**Authors:** Shengli Ma, Lijun Yang, Hui Li, Xinghe Chen, Xiaoyu Lin, Wenyu Ge, Yindong Wang, Liping Sun, Guiping Zhao, Bing Wang, Zheng Wang, Meng Wu, Xin Lu, Muhammad Luqman Akhtar, Depeng Yang, Yan Bai, Yu Li, Huan Nie

**Affiliations:** 1 Heilongjiang Provincial Hospital, Harbin Institute of Technology, Harbin, China; 2 School of Life Science and Technology, Harbin Institute of Technology, Harbin, China; 3 CAS Key Laboratory of Separation Science for Analytical Chemistry, Dalian Institute of Chemical Physics, Chinese Academy of Sciences, Dalian, China

**Keywords:** COVID-19, Metabolomics, Influenza, Metabolic pathways, Oral microenvironment

## Abstract

**Background:**

Coronavirus disease 2019 is a type of acute infectious pneumonia and frequently confused with influenza since the initial symptoms. When the virus colonized the patient's mouth, it will cause changes of the oral microenvironment. However, few studies on the alterations of metabolism of the oral microenvironment affected by SARS-CoV-2 infection have been reported. In this study, we explored metabolic alterations of oral microenvironment after SARS-CoV-2 infection.

**Methods:**

Untargeted metabolomics (UPLC-MS) was used to investigate the metabolic changes between oral secretion samples of 25 COVID-19 and 30 control participants. To obtain the specific metabolic changes of COVID-19, we selected 25 influenza patients to exclude the metabolic changes caused by the stress response of the immune system to the virus. Multivariate analysis (PCA and PLS-DA plots) and univariate analysis (students’ t-test) were used to compare the differences between COVID-19 patients and the controls. Online hiplot tool was used to perform heatmap analysis. Metabolic pathway analysis was conducted by using the MetaboAnalyst 5.0 web application.

**Results:**

PLS-DA plots showed significant separation of COVID-19 patients and the controls. A total of 45 differential metabolites between COVID-19 and control group were identified. Among them, 35 metabolites were defined as SARS-CoV-2 specific differential metabolites. Especially, the levels of cis-5,8,11,14,17-eicosapentaenoic acid and hexanoic acid changed dramatically based on the FC values. Pathway enrichment found the most significant pathways were tyrosine-related metabolism. Further, we found 10 differential metabolites caused by the virus indicating the body’s metabolism changes after viral stimulation. Moreover, adenine and adenosine were defined as influenza virus-specific differential metabolites.

**Conclusions:**

This study revealed that 35 metabolites and tyrosine-related metabolism pathways were significantly changed after SARS-CoV-2 infection. The metabolic alterations of oral microenvironment in COVID-19 provided new insights into its molecular mechanisms for research and prognostic treatment.

**Supplementary Information:**

The online version contains supplementary material available at 10.1186/s12879-022-07979-y.

## Background

Coronavirus disease 2019 (COVID-19) is a novel severe acute respiratory syndrome caused by acute respiratory syndrome coronavirus 2 (SARS-CoV-2) that has a serious impact on global public health. The initial symptoms of COVID-19 patients are similar to influenza, such as fever, cough, fatigue, shortness of breath, and dyspnea [1]. But SARS-CoV-2 could induce variations of cell metabolism and severe acute respiratory syndrome, kidney failure, and even death [2]. The major entry gateways for SARS-CoV-2 are the oral and nasal cavities. When the SARS-CoV-2 invades the oral cavity, it triggers the variations of microenvironment and the alterations of oral metabolism. However, the oral metabolic changes of COVID-19 patients remain unknown until now. As a result, studying the metabolic alterations of oral microenvironment following SARS-CoV-2 infection will provide a new perspective on exploring the metabolic disorders caused by SARS-CoV-2, as well as a novel method of subsequent prevention and treatment [3].

Metabolomics is one of the developing “-omics” technologies, depicting the basic characteristics and main activities of life, which presents a clear and comprehensive description of the internal life activities of organisms and the development state of diseases [4, 5]. When the organism is stimulated by external factors, it will cause a series of small endogenous molecular substances changes. As a consequence, metabolomics can be widely utilized to find the changing features of small molecules, discover biomarkers and molecular mechanisms of disease. Because of the wide application of metabolomics, scientists have carried out a series of studies on COVID-19 metabolomics, such as serum, plasma, urine, exhaled breath, nasopharyngeal swabs, etc. [6–11]. For analysis of serum metabolomics, Shi D found the butyric acid, 2-hydroxybutyric acidand l-glutamic acid were distinctive from those of healthy controls, some of which might be used for predicting severe patients [6]. Shen B noted that massive amino acids and derivatives were significantly decreased in COVID-19 patients compared with healthy controls, which might be used in the selection of potential blood biomarkers [7]. Studies on plasma metabolomics have shown the lipid alterations were significantly correlated with the process of COVID-19, indicating that the development of COVID-19 affected systemic metabolism [8]. The research of urine metabolomics revealed the distinct changes of energy metabolism and purine metabolism in patients with and without acute kidney injury [9]. In addition, Grassin-Delyle S studied the metabolomics of the exhausted breath and discovered the signatures associated with COVID-19, which indicated that the breathprint could distinguish COVID-19 patients from healthy individuals [10]. Liu explored the metabolomics study of nasothroat and discovered two declining metabolites of benzoate and prostaglandin H2 (PGH2), as well as five decreasing metabolic pathways associated with PGH2 in COVID-19 [11]. The above results illustrated the metabolic changes caused by SARS-CoV-2, and also partly involved the body’s regulation of immune function against virus invasion. However, the specific metabolic changes caused by SARS-CoV-2 have not been well studied.

Although some researches have been done to study the relationship between metabolomics and SARS-CoV-2 infection, few studies were performed on the oral cavity [3]. The oral cavity is an excellent source of easy access to biological materials, such as saliva and oral cells, which can be employed in genetics, proteomics, metabolomics, and microbiome research [12–20]. Metabolomics of oral secretion samples collected from pharyngeal mucosa cells can accurately reflect metabolic changes and well explain the relationship between oral microenvironment and SARS-CoV-2 colonization.

Herein, we conducted the metabolomics study on oral secretion samples, including 25 COVID-19 patients and 30 healthy controls. Especially, we selected 25 influenza patients to eliminate the metabolic changes caused by the stress response of the immune system to the virus. Further, we analyzed the specific metabolites and metabolic pathways between COVID-19 and healthy controls and found that there were 35 specific metabolites and 2 tyrosine-related metabolism pathways. In addition, by analyzing the metabolic changes of the body caused by the virus, it was found that 10 differential metabolites were caused by virus-induced changes in body metabolism, and 2 were influenza-specific differential metabolites. All of the findings provided novel insights into the oral metabolic changes impacted by SARS-CoV-2.

## Materials and methods

### Reagents and chemicals

Methanol with HPLC grade were bought from Fisher (Waltham, MA, USA); formic acid and ammonium formate (HPLC grade) were purchased by Sigma-Aldrich (St. Louis, MO, USA); deionized water was produced by a Milli-Q ultrapure water system (Millipore, Billerica, MA, USA).

### Enrollment of participants

All patients were recruited and pathologically confirmed from Heilongjiang Provincial Hospital and this study was approved by the Medical Ethics Expert Committee of Heilongjiang Provincial Hospital for Ethics Review (2020) No. 004. Informed consents were obtained from all the enrolled participants before taking part in this study. The basic clinical information of all the participants recruited in this study is shown in Table 1. The participants were categorized into three groups, 30 healthy controls (Control), 25 influenza patients (Influenza) and 25 COVID-19 patients (COVID-19). The samples of recruiters in the control group were all negative and disease-free samples from the hospital physical examination center. Among all the COVID-19 patients, 23 were mild COVID-19, and the remaining 2 severe patients with one or more of the following criteria: respiratory distress greater than or equal to 30 breaths/min, oxygen saturation no more than 93% at rest, arterial partial pressure of oxygen/fraction of inspired oxygeno more than 300 mmHg or chest imaging showing obvious lesion progression within 24–48 h more than 50% or even organ failure requiring intensive care unit admission [3].

**Table 1:** Demographic details of participants recruitment

Characteristic	COVID-19 (n = 25)	Influenza (n = 25)	Health (n = 30)
Age (year)	46.9 ± 12.3	50.9 ± 20.4	40.7 ± 13.2
Sex (M/F)	15/10	19/6	13/17

Statistical description of age was presented as Mean ± Standard Deviation

### Sample collection

The sample collection time of patients with COVID-19 was 4–11 days after admission. First of all, the recruits were gargled with clean water for three times, then a doctor applied disposable sterile swabs by swipping three to five times to collect mucosal cells of the posterior pharynx, lateral wall, and crypts of the tonsil of the participants.Then, the swabs were placed into oral swab preservation tubes (purchased from Kangwei Century Biotechnology Co., Ltd.) and stored at 4 °C for next procedure of metabolites extraction. Quality control samples (QCs) were prepared by mixing all the tested samples in equal quantities, which provided a mesasurement of stability and performance of the system.

### Metabolites extraction

After taking out the throat swab samples stored at 4 °C, soaking in 1.5 mL methanol for 20 min, the samples were centrifuged at 25,000 rpm for 15 min at 4 °C, 600 μL of the supernatant was collected into a new EP tube and stored at − 80 °C, for further analysis.

### UPLC-MS analysis

The experiment was performed with a Waters Acquity™ ultra-performance liquid chromatography (UPLC) system (Waters, Milford, MA, USA) coupled with a Q Exactive mass spectrometer equipped with a dual electrospray ion source (Thermo Fisher Scientific, USA) with BEH C18 (2.1 mm × 100 mm, 1.7 µm) column (Waters, Milford, USA) operated in the positive (ESI^+^) and negative (ESI^−^) mode. In positive mode, the mobile phase consisted of A (water with 0.1% formic acid) and B (methanol with 0.1% formic acid). In negative mode, the mobile phase consisted of A (water with 10 mM ammonium formate) and B (methanol with 10 mM ammonium formate).The optimized UPLC elution gradient was set as follows: 2% B for the initial 1.0 min, 2–98% B from 1 to 9 min, maintenance at 98% B from 9 to 12 min, progression of the gradient back to 2%B from 12 to 12 min and finally maintenance at 2%B from 12.1 to 15 min in both positive and negative mode. The flow rate was set at 0.3 mL/min, with the temperature of the autosampler of 4 °C. The volume was injected 5 μL for each run, and the column temperature was maintained at 45 °C.

Data acquisitions including MS acquisition and MS/MS identification were collected by Q exactive mass spectrometer (Thermo Fisher Scientific, USA). The mass scan range was from 70 to 1050 m/z with the max injection time 100 ms. For MS/MS analysis, the collision energy (CE) used was ranged from 20 to 60 eV as a function of molecular weight (MW) with the max injection time 50 ms. The capillary temperature was 320 °C and Aux gas heater temperature was 350 °C.

### Data processing

Data pre-processing, including peak detection, noise filtering, feature alignment and data normalization [Probabilistic Quotient Normalization (PQN) method] were performed by using the XCMS package in R-project platform. The parameters were applied as follows: the bandwidth was set at 15 s, and the peak width was ranged from 5 to 30 s; other parameters are selected as default values.

### Compound identification

For metabolites identification, the process was based on compound identification principles proposed by MSI (Metabolomics Standards Initiative, proposed by the Association of Metabolomics) in 2007. The structural information was firstly matched in databases for m/z and MS/MS spectrum analysis, including multiple databases such as BGI Library (BGI self-built standard library), mzCloud and ChemSpider (HMDB (Human Metabolome Database (HMDB, http://www.hmdb.ca/), KEGG (Kyoto Encyclopedia of Genes and Genomes, https://www.kegg.jp/), LipidMaps (https://www.lipidmaps.org/)) multiple databases. According to the information available for matching (including primary molecular weight, secondary fragmentation spectrum, column retention time, presence or absence of reference standards, etc.), the identified substances are annotated with confidence levels, thereby the confidence level of the annotation selected in the study were level 1 (substances that can be accurately identified based on standard database and experimental data) and level 2 (substances whose structural formula can be matched to a standard database).

### Statistical analysis

Principal component analysis (PCA) was conducted to provide a measurement of the stability and performance of the system. Partial least squares discriminant analysis (PLS-DA) was employed to characterize the global alterations of COVID-19, influenza and control groups. Before establishing PCA and PLS-DA models, log2 transformation was performed on the data, and Pareto scaling was used to scale the data. Meanwhile, cross-validation was conducted to guarantee the stability and credibility of the PLS-DA models to avoid overfitting. Furthermore, the Student’s t-test was performed to characterize the differential metabolites. Heatmap was created to visualize the clustering and individual discrete trend among the groups. Pathway analysis was operated to clearly characterize the main metabolic pathways of differential metabolites mapping.

The PCA, PLS-DA and cross-validation were performed by using MetaboAnalyst (https://www.metaboanalyst.ca/MetaboAnalyst/) and R-project platform. The Student’s t-test was created by using GraphPad Prism 8.0 (GraphPad Software, USA). Heatmap and pathway analysis were conducted with the on-line based Hiplot (https://hiplot.com.cn/) and MetaboAnalyst.

## Results

### Metabolic profiles of oral secretion samples in health, influenza and COVID-19

**Fig. 1:**
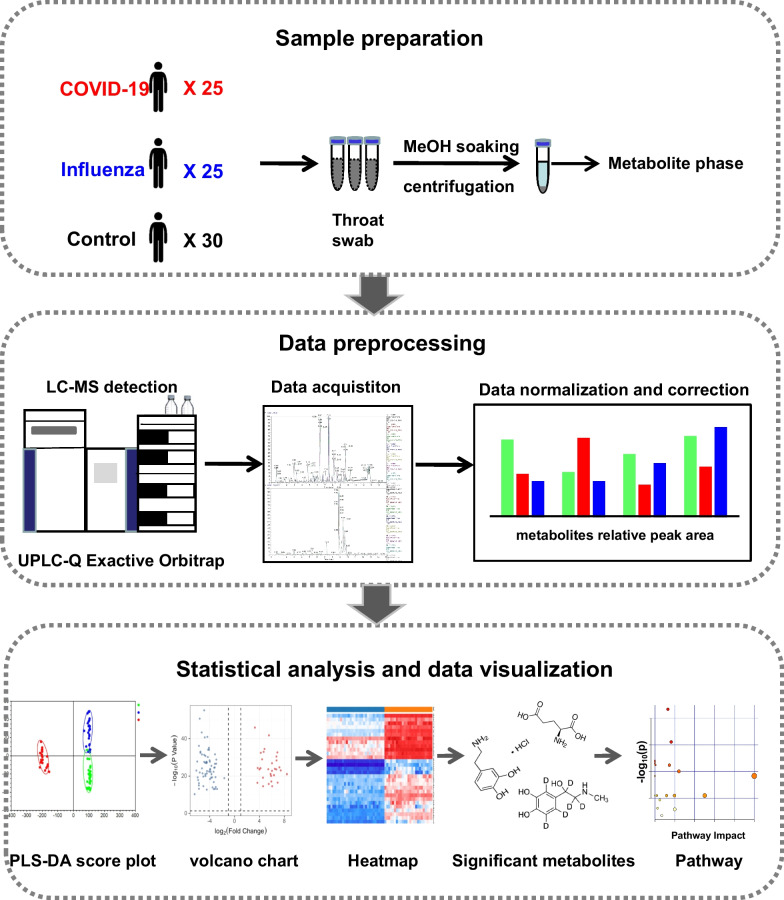
The workflow for data analysis of oral metabolomics of COVID-19

**Fig. 2:**
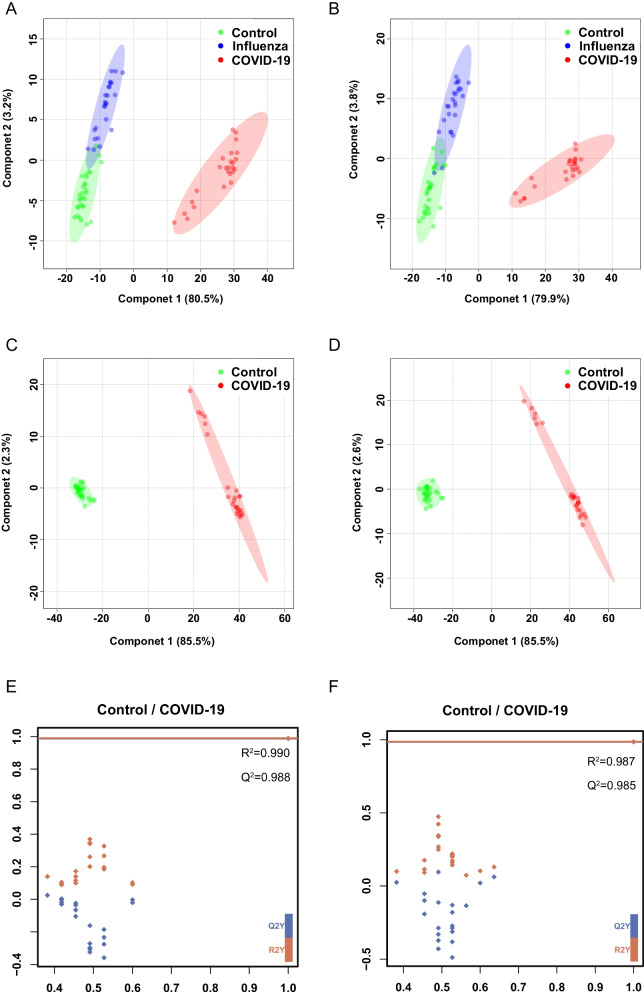
PLS-DA score plot and cross-validation plot of metabolic profiling analysis. **A** PLS-DA score plot of Control, Influenza and COVID-19 group in positive mode. Green nodes: Control subjects, blue nodes: Influenza subjects, red nodes: COVID-19 subjects. **B** PLS-DA score plot of Control, Influenza and COVID-19 group in negative mode. Green nodes: Control subjects, blue nodes: Influenza subjects, red nodes: COVID-19 subjects. **C** PLS-DA score plot of Control and COVID-19 group in positive mode. Green nodes: Control subjects, red nodes: COVID-19 subjects. **D** PLS-DA score plot of Control and COVID-19 group in negative mode. Green nodes: Control subjects, red nodes: COVID-19 subjects. **E** Cross-validation plot in positive mode. **F** Cross-validation plot in negative mode

UPLC-Q Exactive Orbitrap-MS analysis was used to analyze 80 oral secretion samples to investigate whether the oral metabolites differ from participants of COVID-19, influenza and control. The overall design of the study was depicted in Fig. 1. The base peak chromatogram (BPC) had a good resolution in positive and negative ion patterns and significant differences of the three groups (Additional file 1: Fig.S1). To visually reflect the overall metabolic profiling differences and similarities, the partial least squares discriminant analysis (PLS-DA) were employed for the three groups. The PLS-DA (Fig. 2A, B) results displayed a clear separation of COVID-19 patients from the other two groups in both positive and negative mode. In PLS-DA plots, the COVID-19 group was on the left, while control and influenza groups were on the right, with a closer tendency. To further visualize the specific differences between COVID-19 patients and control, we re-established PLS-DA plot and found the remarkable separation between COVID-19 and control group (Fig. 2C, D). In addition, the cross-validation test showed high predictability and goodness-of-fit values of the model as indicated by R^2^Y and Q^2^Y (R^2^Y = 0.990, Q^2^Y = 0.988 in positive mode and R^2^Y = 0.987, Q^2^Y = 0.985 in negative ion mode) (Fig. 2E, F). Through 100 permutation tests, the p value was less than 0.01, and the F value were 15,878 and 43,460, respectively (Additional file 1: Fig.S2A-B). Moreover, there were no significant differences in age, gender and ethnicity among the three groups of patients (data not shown). In addition, the COVID-19 patients have not been vaccinated, and mainly received antiviral, antibiotic and adjuvant drug treatment. However, by comparing the differences between COVID-19 patients and COVID-19 patients with drug treatment (Tre-COVID-19), found that drug treatment did not cause more significant metabolic differences between the two groups (Additional file 1: Fig.S3, Table S1).

### Metabolic analysis of oral secretion samples in COVID-19 and health

**Fig. 3:**
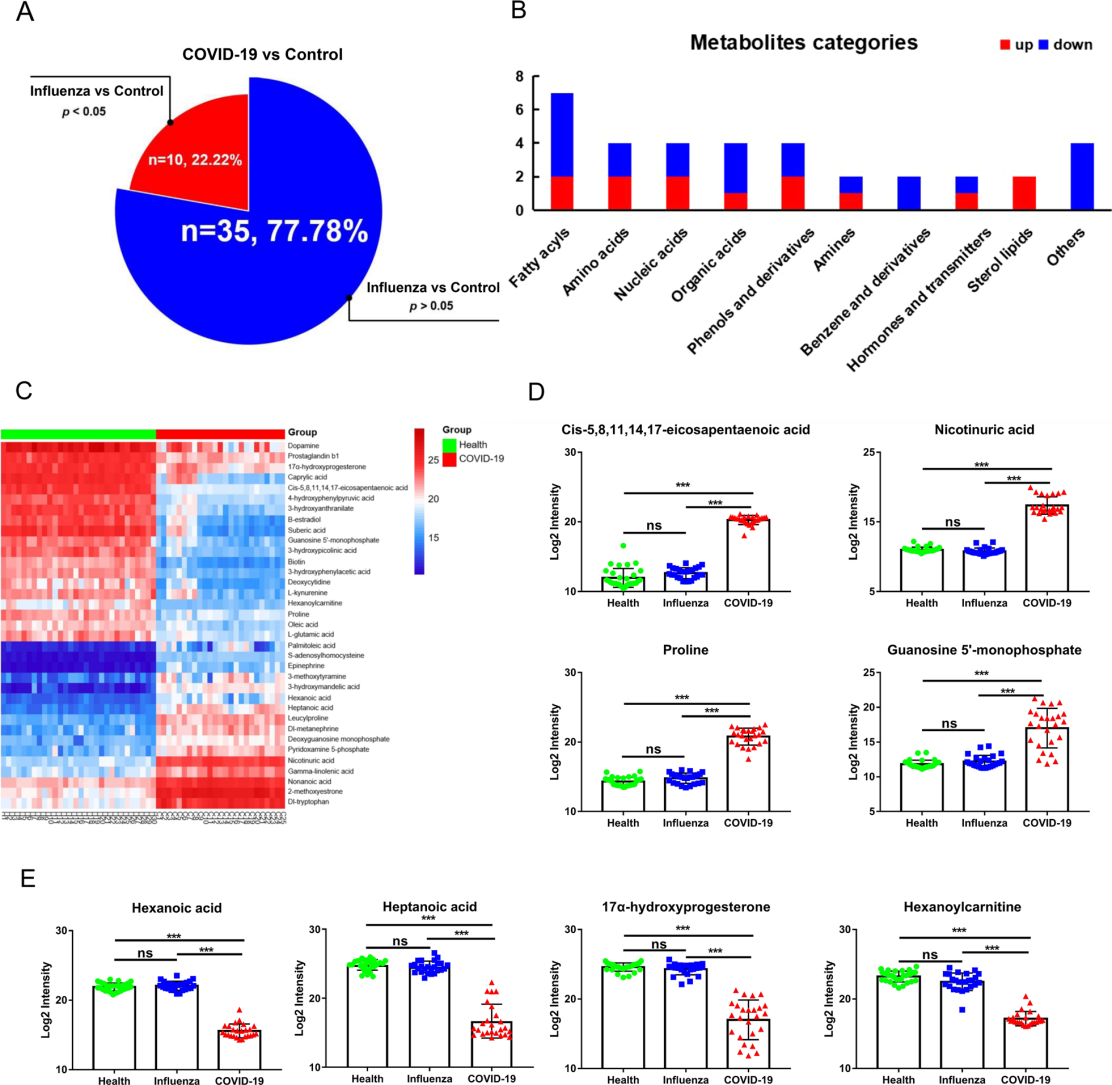
Identification, screening and classification of metabolites between COVID-19 and control group. **A** The venn diagrams of the different features obtained with VIP > 1, p < 0.5 and FC ≥ 1.2 or ≤ 0.83 in different groups, 35 metabolites showed no significant differences in control and influenza, 10 metabolites showed significant differences in control and influenza. **B** Classification donut chart of 35 identified differential metabolites. **C** The heatmap of 35 metabolites dramatically changed in COVID-19 and control. **D** The box plots of cis-5,8,11,14,17-eicosapentaenoic acid, nicotinuric acid, guanosine 5′-monophosphate and proline based on the FC value greater than 100 in COVID-19. Green: Control subjects, blue: Influenza subjects, red: COVID-19 subjects. **E**. The box plots of hexanoic acid, heptanoic acid, 17α-hydroxyprogesterone and hexanoylcarnitine were screened out based on the FC value less than 0.02 in COVID-19. Green: Control subjects, blue: Influenza subjects, red: COVID-19 subjects

Further, the univariate and multivariate analysis methods were employed to obtain specific differential metabolites. 45 metabolites were screened between COVID-19 and control group with VIP scores values greater than 1.0, the p values less than 0.05, and fold change values greater than or equal to 1.2 or no more than 0.83 (Fig. 3A, Additional file 1: Table S2). As influenza is also a viral infection, which may cause metabolic changes in the body, the influenza group was selected to eliminate the metabolic changes caused by the stress response of the immune system to the virus. Therefore, we found 35 metabolites with no differences between influenza and control groups, which indicated that these were COVID-19 specific differential metabolites. Moreover, 35 specific metabolites were classified, mainly including amines and derivatives, amino acids, benzene and derivatives, hormones and transmitters, fatty acyls, nucleic acids, organic acids, phenols and derivatives, sterol lipids and others, in which the identified benzene and derivatives all decreased and sterol lipids increased (Fig. 3B). To observe the overall variation of the metabolites, a heatmap based on the identified 35 metabolites was produced and showed a good result of clustering and individual discrete trend of the COVID-19 patients and control group (Fig. 3C). Moreover, according to the distribution analysis of 35 differential metabolites, 16 differential metabolites increased and 19 decreased in the COVID-19 group compared with the control or influenza. Among the rising metabolic species, cis-5,8,11,14,17-eicosapentaenoic acid, nicotinuric acid, guanosine 5′-monophosphate and proline were screened out based on the FC value greater than 100 and hexanoic acid, heptanoic acid, 17α-hydroxyprogesterone and hexanoylcarnitine were screened out based on the FC value less than 0.02 in the declining metabolites (Fig. 3D, E).

### Abnormal metabolic pathways in COVID-19, especially tyrosine-related metabolism pathway

**Fig. 4:**
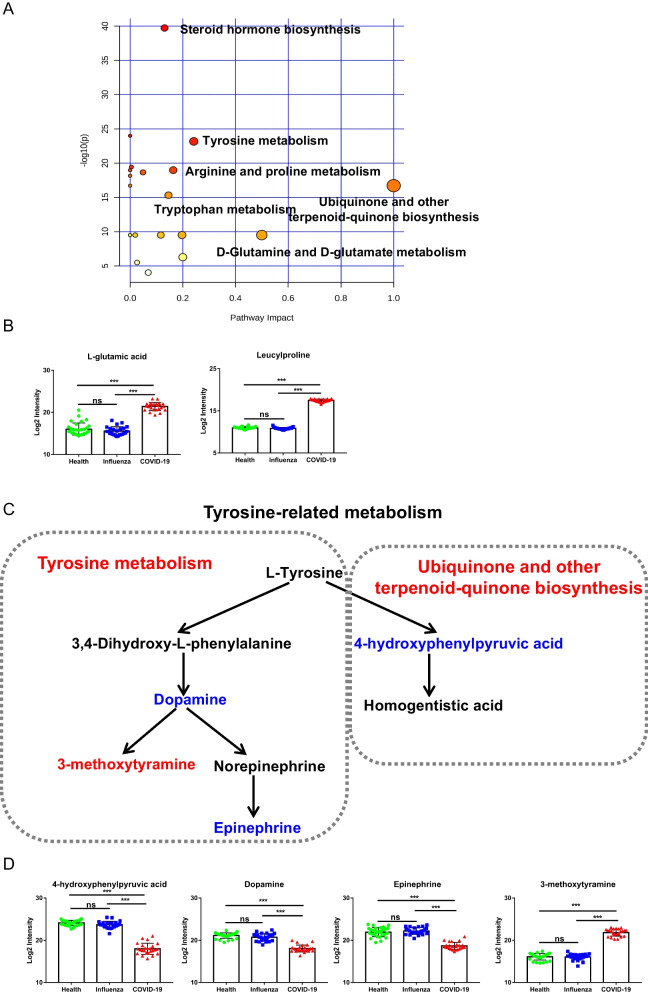
Metabolic pathways in COVID-19, especially tyrosine-related metabolism pathway. **A** Metabolic topological analysis diagram of COVID-19 metabolomics. **B** The box plots of L-glutamic acid, proline and leucylproline in control, influenza and COVID-19. Green: Control subjects, blue: Influenza subjects, red: COVID-19 subjects. **C** The interactive network of tyrosine-related metabolic pathways. **D** The box plots of 4-hydroxyphenylpyruvic acid, dopamine, epinephrine, and 3-methoxytyramine in control, influenza and COVID-19. Green: Control subjects, blue: Influenza subjects, red: COVID-19 subjects. *p < 0.05, **p < 0.01, *** p < 0.0001

The metabolic pathway analysis was further carried out through Metaboanalyst 5.0 website. Then 17 metabolic pathways were matched through KEGG database as disturbed in oral metabolic profiles of COVID-19 patients. Interestingly, most of these dysfunctional pathways were mainly focused on amino acid metabolisms, such as arginine and proline metabolism, tryptophan metabolism, tyrosine metabolism and some related pathways (Fig. 4A). In our metabolomics data, there were 4 amino acids and derivatives changed remarkably including nicotinuric acid mentioned above. The levels of l-glutamic acid, proline and leucylproline illustrated a noticeable increased trend in COVID-19 compared with the control group (Fig. 4B). Moreover, according to the conditions of -log (P) value > 15 and path impact > 0.2, 2 main metabolic pathways were obtained, including Ubiquinone and other terpenoid-quinone biosynthesis and tyrosine metabolism. Interestingly, the 2 metabolic pathways were related to tyrosine metabolism. We mapped an interactive network of tyrosine-related metabolic pathways (Fig. 4C). 4 differential metabolites were obtained in our data, included 4-hydroxyphenylpyruvic acid, dopamine, epinephrine, and 3-methoxytyramine, of which only 3-methoxytyramine showed an upward trend compared with the control group, and the rest showed a downward trend (Fig. 4D).

### Metabolic changes of the body caused by influenza virus

**Fig. 5:**
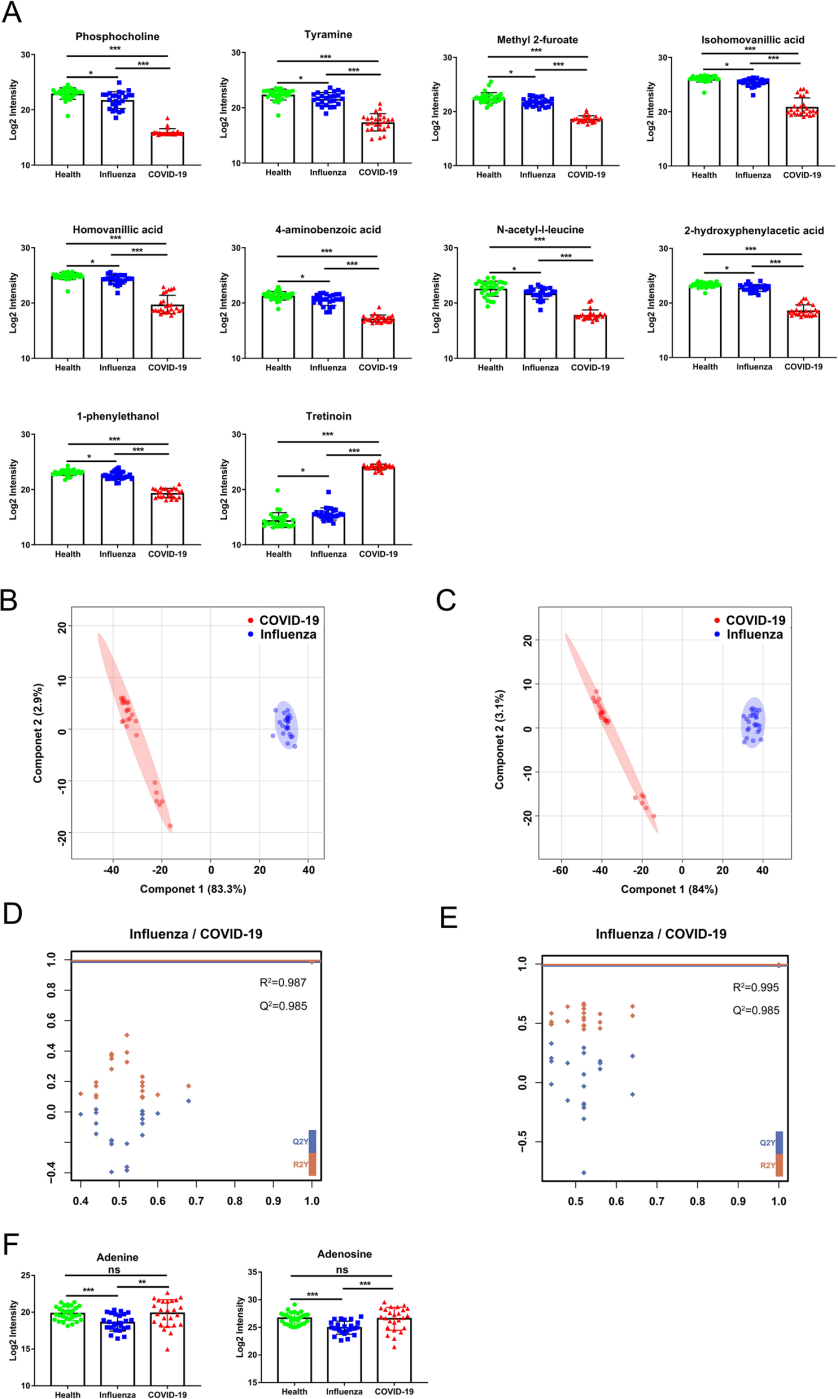
Changes in metabolites caused by the body's feedback to the virus. **A** Relative amounts of 10 metabolites in control, influenza and COVID-19 might reflect the body’s feedback after virus infection. **B** PLS-DA score plot of Influenza and COVID-19 group in positive mode. Blue nodes: Influenza subjects, red nodes: COVID-19 subjects. **C** PLS-DA score plot of Influenza and COVID-19 group in negative mode. Blue nodes: Influenza subjects, red nodes: COVID-19 subjects. **D** Cross-validation plot in positive mode. **E** Cross-validation plot in positive mode. **F** Relative amounts of 2 metabolites significantly decreased in Influenza group. *p < 0.05, **p < 0.01, ***p < 0.0001

According to the above-established PLS-DA model of COVID-19 and control, we obtained 10 differential metabolites that were also significantly different between influenza and control, which might reflect the response to the body’s stimulation by external viruses (Fig. 3A). Notably, 9 of 10 metabolites continued to decline in control, influenza and COVID-19, and they were 1-phenylethanol, isohomovanillic acid, methyl 2-furoate, N-acetyl-l-leucine, phosphocholine, tyramine, 2-hydroxyphenylacetic acid, 4-aminobenzoic acid, homovanillic acid (Fig. 5A). Only tretinoin continued to rise in control, influenza and COVID-19 (Fig. 5A). These results proved that the changes of these differential metabolites caused by COVID-19 were more prominent compared with influenza group.

Since the early symptoms of COVID-19 were similar to those of influenza, we further analyzed the metabolic differences between influenza and COVID-19. PLS-DA plots (Fig. 5B, C) shows that the two groups of samples were obvious aggregation within the group and dispersion in two different regions between the groups, without overfitting in the permutation test, which was repeated 100 times (Fig. 5D, E, Additional file 1: Fig.S2C-D). It was worth noting that there were two metabolites, adenine and adenosine, that showed the lowest trend in the influenza group, and there was no significant difference between COVID-19 and control group (Fig. 5F). They specifically expressed the metabolic changes caused by influenza infection.

## Discussion

In this study, we first constructed model of COVID-19 and control and carried out pathway enrichment. A total of 35 COVID-19 specific differential metabolites were identified, of which 16 differential metabolites increased and 19 decreased in COVID-19 group compared with control group or influenza participants. And the levels of cis-5,8,11,14,17-eicosapentaenoic acid and hexanoic acid changed dramatically based on the FC value. It has been reported that human umbilical cord blood vessels can convert cis-5,8,11,14,17-eicosapentaenoic acid into prostaglandin I3 with anti-inflammatory effect, which can reduce the symptoms of dysmenorrhea [21, 22]^.^ Our results showed that cis-5,8,11,14,17-eicosapentaenoic acid accumulated significantly in COVID-19, which proved that there was a serious inflammatory reaction in the COVID-19 patients. The research results found that hexanoic acid could promote the differentiation of TH1 and TH17 lymphocytes, and the activation of these two cells was related to inflammation, so hexanoic acid can support inflammation through the above functions [23]. We found that hexanoic acid decreased significantly in COVID-19, indicating that most of hexanoic acid was secreted into the blood to promote the inflammatory response, while hexanoic acid in oral cells decreased dramatically.

The pathway enrichment results showed that metabolic pathways based on 35 different metabolites between COVID-19 and the control group were focused on the amino acid metabolism, and the main pathways were tyrosine-related pathways, which targeted dopamine and 4-hydroxyphenylpyruvic acid (HPPA). As a non-essential amino acid, tyrosine is obtained through the hydroxylation reaction of phenylalanine and food [24]. In one of the pathways of tyrosine oxidation, part of tyrosine is metabolized to dopamine, and then decomposed into 3-methoxytyramine and epinephrine. Further studying the dysfunctional pathways, we found dopamine was remarkably decreased in patients with the infection of SAR-CoV-2. Rodrigo Arreola et al. reported that the decrease of dopamine content would lead to the decrease of human immune function [25]. Kenneth Blum’s study found that dopamine synthesis might decrease in COVID-19, because SARS-Cov2 would induce down-regulation of angiotensin I converting enzyme 2 (ACE2) gene expression and the coexpression gene of dopa decarboxylase [26]. Therefore, the decreased dopamine might cause the disordered immune system through ACE2 receptors. In another path of tyrosine oxidation, HPPA can be converted from phenylalanine through tyrosine, and then HPPA is oxidized by 4-hydroxyphenylpyruvate dioxygenase to homogentisic acid, final generation coenzyme A and fumaric acid [27]. Coenzyme A and fumaric acid enter the tricarboxylic acid cycle and participate in important energy metabolism pathways in organisms [28, 29]. Luporini et al. found that the content of HPPA and phenylalanine were both decreased in mild COVID-19 patient serum [30]. Our results also showed the level of HPPA was lower in the oral cavity of COVID-19 patients, despite no change in control and influenza participants, implying that the decreased level of HPPA in tyrosine pathway might affect the energy metabolism of patients.

According to the feedback of the body to the virus, we screened 10 differential metabolites caused by the virus, including phosphocholine, tyramine and N-acetyl-l-leucine and so on. Phosphocholine is the main phospholipid component in eukaryotic membranes and exists in symbionts or pathogenic bacteria associated with eukaryotes in prokaryotes. It has been reported to exhibit surprising immunomodulatory properties [31]_._ Our results showed that phosphocholine continuously decreased in control, influenza and COVID-19, which might suggest phosphocholine turned on the immune regulation of virus when the body received the stimulation of external virus. Tyramine, a derivative of tyrosine, has been shown to act as a catecholamine releaser in humans. Low levels of tyramine can lead to the pro-inflammatory state of MetS (Background Metabolic syndrome) [32]. In this research, we found tyramine showed a persistent decrease in the three groups, which might indicate the degree of inflammatory response presented by the body after receiving influenza and SARS-CoV-2, respectively, and the inflammatory response caused by SARS-CoV-2 was more intense. N-acetyl-l-leucine is a potent endogenous metabolite, and studies have shown that after oral administration of N-acetyl-l-leucine in mice, pro-inflammatory cytokines in the cortex was significantly reduced, thereby reducing traumatic brain injury inflammatory response [33]. The results of this study showed that N-acetyl-l-leucine was persistently decreased in the three groups, indicating that SARS-CoV-2 caused a more severe inflammatory response in the body.

In response to the feedback of influenza virus to the body, we screened out adenine and adenosine that were significantly reduced in influenza patients compared with the COVID-19 and control group. Adenine is a purine that is one of the four bases in DNA nucleic acid and is the chemical constituent of DNA and RNA. Adenine plays an important role in cellular respiration, formation of ATP, the cofactors NAD and FAD, and protein synthesis [34, 35]_._ And adenosine is an endogenous nucleoside that spreads all over human cells. It can directly enter the myocardium and phosphorylate to produce adenylate, which is involved in myocardial energy metabolism [36, 37]. In immune cells, adenosine is ubiquitous and modulates inflammatory responses by interacting with AR, one of the subtypes of G protein-coupled receptors. For example, enhances lymphocyte function, thereby enhancing immune regulation. Various studies have shown that AR can enhance the body’s immune function by increasing adenosine levels [38]. Our results show that adenosine in the influenza group has a significant decrease compared with the other two groups, proving that the influenza virus caused a decline in the body’s immune function. These findings might indicate the changes of immune function of body and energy metabolism caused by influenza virus stimulation.

Taken together, the metabolomics study on oral secretion samples revealed the distinct metabolic changes of oral microenvironment in patients after infection of SARS-CoV-2. We have identified 35 metabolites with significant differences between COVID-19 and control, and found that tyrosine-related pathways reflect the major dysfunctional pathways in the oral microenvironment after infection with SARS-CoV-2. Further, we analyzed the metabolic differences between influenza and COVID-19, and found that adenine and adenosine caused the most obvious body response after influenza virus infection. This research revealed the oral metabolic signatures of COVID-19 patients that were more likely to reflect the features of metabolic reprogramming in body cells, which could provide valuable information to the deep study on molecular mechanisms and lay the foundation for treatment.

## Conclusions

Through non-targeted metabolomics analysis, 35 specific differential metabolites were defined as SARS-CoV-2 specific differential metabolites, which were no differences between influenza and control group. Pathway analysis showed tyrosine-related metabolism were most dysfunctional in COVID-19. The oral metabolomics study revealed the characteristics of metabolic alterations in oral microenvironment in COVID-19 and provided new insights for research and prognostic treatment.

## Supplementary Information

**Additional file 1.** Supplementary figures.

**Additional file 2.** Raw data.

## Data Availability

All data generated or analysed during this study are included in this published article [and its Additional file 2 files named Raw data].

## Abbreviations

COVID-19: Coronavirus disease 2019
SARS-CoV-2: Acute respiratory syndrome coronavirus 2
UPLC: Ultra-performance liquid chromatography
BPC: Base peak chromatogram
PCA: Principal component analysis
PLS-DA: Partial least squares discriminant analysis
RT: Retention time
m/z: Mass-to-charge ratio
FC values: Fold change values
PGH2: Prostaglandin H2
ARDS: Acute respiratory distress syndrome
ACE2: Angiotensin I converting enzyme 2
HPPA: 4-Hydroxyphenylpyruvic acid
