# Supplementary material for: Understanding metabolic alterations after SARS-CoV-2 infection: insights from the patients’ oral microenvironmental metabolites
Source: BMC Infect Dis. 2023 Jan 23;23:42. doi: 10.1186/s12879-022-07979-y (PMC9869582; doi:10.1186/s12879-022-07979-y)
Supplement: Supplementary file 1 — Additional file 1. Supplementary figures. [file 12879_2022_7979_MOESM1_ESM.pdf]

Supplementary figures

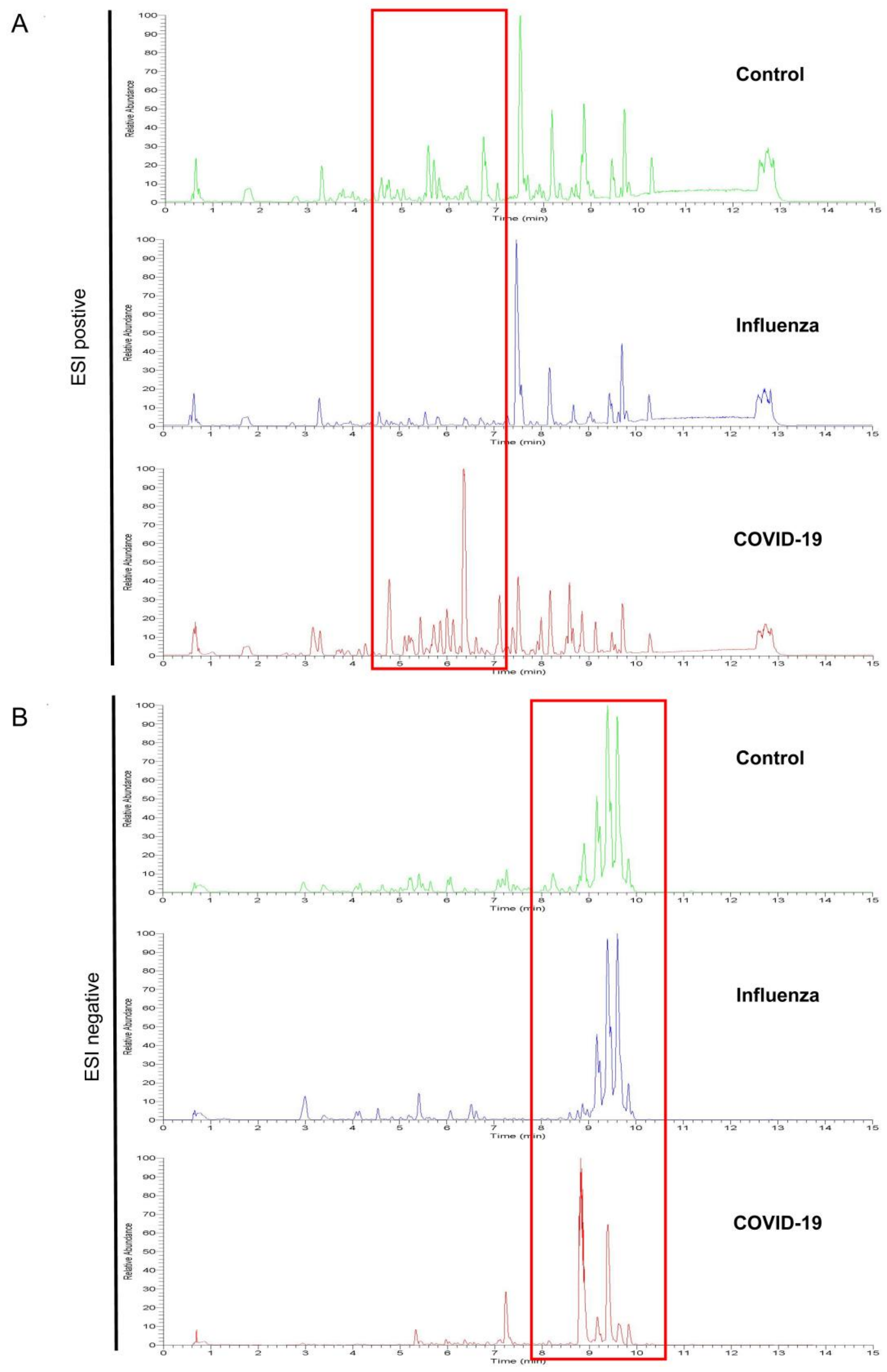

FigS1. The typical BPC chromatographies of Control, Influenza and COVID-19 metabolic profiles in ESI positive and negative mode

A. Control, Influenza and COVID-19 in positive mode.

B. Control, Influenza and COVID-19 in negative mode.

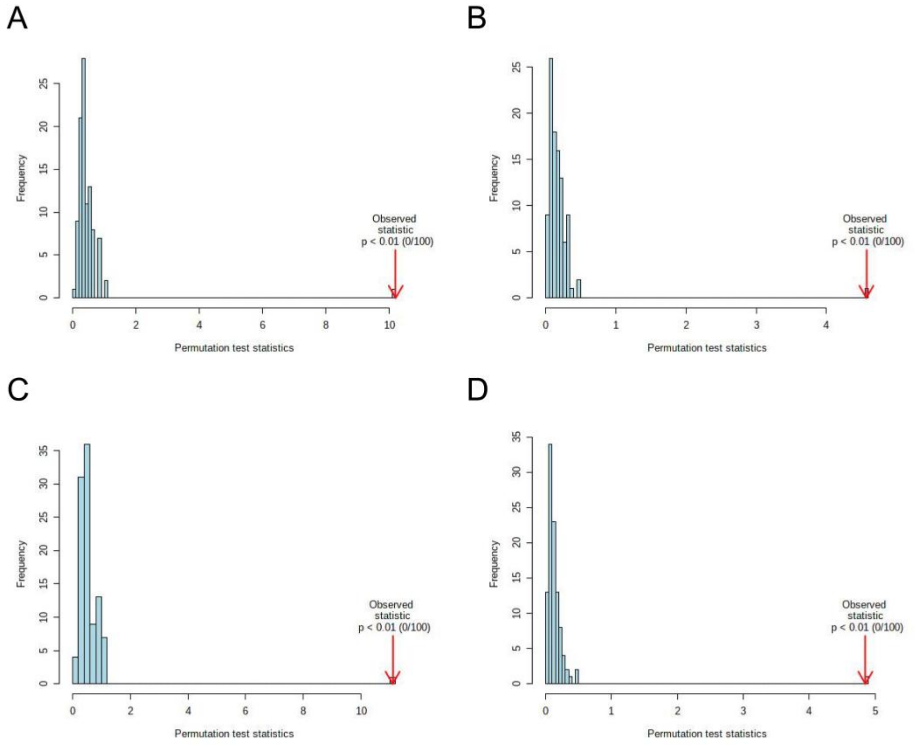

FigS2. Statistical significance of 100 permutation tests

A. Statistical significance of 100 permutation tests between COVID-19 and control in positive mode.

B. Statistical significance of 100 permutation tests between COVID-19 and control in negative mode.

C. Statistical significance of 100 permutation tests between COVID-19 and influenza in positive mode.

D. Statistical significance of 100 permutation tests between COVID-19 and influenza in negative mode.

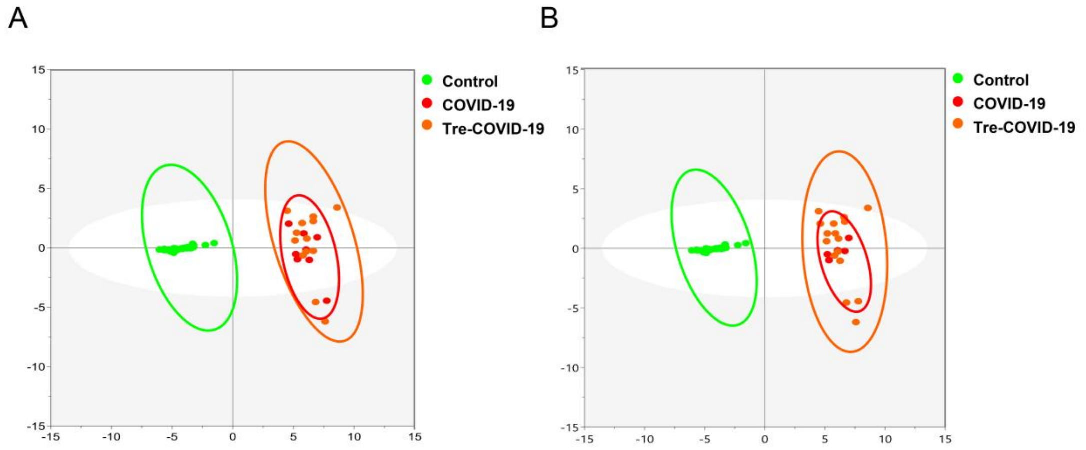

FigS3. Effect of drug treatment on differential metabolites between COVID-19 and control

A. PCA score plot of Control, COVID-19 and Tre-COVID-19 based on antibiotic treatment.

B. PCA score plot of Control, COVID-19 and Tre-COVID-19 based on adjuvant treatment.

Supplementary table lists

Table S1 Summary of medication for patients with COVID-19

	**Antiviral therapy**						**Antibiotic therapy**		**Adjuvant therapy**					
No.	Arbidol	Oseltamivir	triazavirin	LopinavirandRitonavir	Ribavirin	Interferon	Cephalosporins	Moxifloxacin	Bromhexine	Ambroxol	Amino acid	Vitamin	Albumin	Immunoglobulin
1	√	√		√	√	√		√	√		√		√	
2		√		√	√	√		√	√					
3	√				√	√		√			√			
4				√	√	√								
5				√	√	√		√					√	
6			√	√	√	√								
7	NA	NA	NA	NA	NA	NA	NA	NA	NA	NA	NA	NA	NA	NA
8				√	√	√			√		√			
9			√	√	√	√		√	√					
10	NA	NA	NA	NA	NA	NA	NA	NA	NA	NA	NA	NA	NA	NA
11	√			√	√	√		√	√		√			
12			√	√	√	√								
13					√	√	√		√					
14				√	√	√				√				
15		√		√	√	√		√	√		√	√		
16	NA	NA	NA	NA	NA	NA	NA	NA	NA	NA	NA	NA	NA	NA
17	√	√		√	√	√		√	√		√		√	
18				√	√	√							√	
19				√	√	√								
20				√	√				√					
21	NA	NA	NA	NA	NA	NA	NA	NA	NA	NA	NA	NA	NA	NA
22	√	√		√	√	√	√	√	√	√	√	√	√	√
23	√	√		√	√	√		√	√		√	√	√	
24	NA	NA	NA	NA	NA	NA	NA	NA	NA	NA	NA	NA	NA	NA
25	√			√	√	√		√						

Table S2 Summary of the differentialy expressed metabolites in COVID-19 patients relative to healthy controls

Metabolite	VIP	FC	*p*-value	m/z	RT
Tretinoin	1.6599	341.6012	3.88333E-13	300.2093	9.669
Cis-5,8,11,14,17-eicosapentaenoic acid	1.5269	170.6647	5.15566E-13	302.2247	10.038
Nicotinuric acid	1.3227	121.1716	6.26662E-05	180.0536	4.889
Guanosine 5'-monophosphate	1.3169	118.0951	0.001786313	363.0578	1.064
Proline	1.6102	105.5101	1.12796E-07	115.0634	8.903
Leucylproline	1.3428	92.4395	1.1617E-16	228.1476	5.787
Deoxycytidine	1.534	66.1611	3.31143E-12	454.1808	6.358
2-methoxyestrone	1.5554	60.1318	4.06932E-16	300.1725	8.105
3-methoxytyramine	1.5127	54.8292	2.16881E-09	167.0947	3.171
Deoxyguanosine monophosphate	1.2842	45.8606	0.000379024	347.063	0.98
L-kynurenine	1.3506	44.7689	2.52134E-05	208.0849	4.705
Oleic acid	1.207	42.8361	2.22808E-09	282.256	10.329
L-glutamic acid	1.3899	24.0615	2.9118E-07	147.0532	0.77
Prostaglandin b1	1.0842	18.0484	1.01301E-06	336.2306	7.857
Palmitoleic acid	1.0281	15.9615	2.01151E-07	254.2249	9.89
3-hydroxypicolinic acid	1.0044	13.1114	7.37967E-12	139.0269	4.982
Dl-metanephrine	1.2153	12.732	6.36302E-10	197.1053	2.618
Dopamine	1.0885	0.1308	1.13865E-12	153.079	3.491
Pyridoxamine 5-phosphate	1.1324	0.1192	9.57938E-17	248.0574	5.936
Gamma-linolenic acid	1.0996	0.1109	4.45485E-11	278.2245	7.867
Dl-tryptophan	1.2787	0.1099	1.74614E-09	408.1759	4.384
3-hydroxyphenylacetic acid	1.1094	0.1027	1.63108E-09	152.0474	3.525
Epinephrine	1.0703	0.098	8.76548E-08	183.0897	3.427
1-phenylethanol	1.2113	0.086	2.2765E-14	122.0732	5.097
S-adenosylhomocysteine	1.0422	0.0736	3.9405E-06	384.1208	6.508
Biotin	1.2667	0.058	8.47808E-07	244.0884	4.085
4-aminobenzoic acid	1.0828	0.0576	5.9323E-11	137.0477	3.812
Suberic acid	1.1331	0.057	3.61034E-06	174.0891	1.326
Isohomovanillic acid	1.4459	0.0564	9.57938E-17	182.0581	3.644
Methyl 2-furoate	1.2655	0.0549	9.65828E-05	126.0318	4.353
Homovanillic acid	1.2118	0.0539	6.26488E-16	182.0581	3.695
2-hydroxyphenylacetic acid	1.1558	0.0502	9.57938E-17	152.0473	3.972
Tyramine	1.4023	0.0457	8.44363E-12	137.0842	2.997

3-hydroxymandelic acid	1.3313	0.0453	1.3156E-14	168.0422	2.731
N-acetyl-l-leucine	1.3502	0.0349	7.90658E-08	173.1054	3.11
B-estradiol	1.4549	0.0308	4.18844E-14	272.1775	6.29
3-hydroxyanthranilate	1.5024	0.028	1.13865E-12	153.0427	4.049
Nonanoic acid	1.2512	0.0256	4.53215E-14	158.1307	6.505
Caprylic acid	1.3782	0.022	1.3156E-14	144.115	4.807
4-hydroxyphenylpyruvic acid	1.3146	0.0206	8.91161E-14	180.0423	4.402
17α-hydroxyprogesterone	1.5328	0.0188	1.25413E-14	330.2194	7.127
Hexanoylcarnitine	1.5674	0.0188	3.32259E-11	259.1784	3.653
Heptanoic acid	1.4951	0.0163	1.63289E-12	130.0994	4.217
Hexanoic acid	1.309	0.0156	8.91161E-14	116.0838	7.883
Phosphocholine	1.6987	0.0077	3.32259E-11	366.1315	4.064

Variable importance in the projection (VIP) was obtained from PLS-DA model with value greater than 1.0.

Fold change (calculated by dividing the expression mean of disease samples by the expression mean of normal samples) was greater than or equal to1.2 or no more than 0.83.

The *p*-value was calculated from two-tailed student’s test with value lower than 0.05.

m/z was the mass-to-charge ratios of the peaks.

RT was the retention times of the peaks.
